# Transarterial Chemoembolization with Anthracyclines-Loaded Polyethylene Glycol Drug Eluting Microspheres for the Treatment of Hepatocellular Carcinoma: A Pooled Multicentric Analysis of Survival in 580 Patients

**DOI:** 10.1007/s00270-023-03362-9

**Published:** 2023-02-10

**Authors:** Filipe Veloso Gomes, Thierry de Baère, Gontran Verset, Élia Coimbra, Gerardo Tovar-Felice, Katerina Malagari, Jordi Bruix

**Affiliations:** 1 Interventional Radiology Unit, Hospital Curry Cabral, Centro Hospitalar Universitário de Lisboa Central, Lisbon, Portugal; 2 NOVA Medical School/Faculdade de Ciências Médicas, Universidade Nova de Lisboa, Lisbon, Portugal; 3 Departement d’Anesthésie, de Chirurgie, Et de Radiologie Interventionnelle, Gustave Roussy, 102 rue Edourad Vaillant, Villejuif, France; 4 Université Paris-Saclay, UFR Médecine Le Kremlin-Bicêtre, Le Kremlin Bicêtre, France; 5 Centre d’Investigation Clinique BIOTHERIS, INSERM CIC1428, 102 rue Edourad Vaillant, Villejuif, France; 6 Hôpital Erasme, Université Libre de Bruxelles, Brussels, Belgium; 7 Vascular and Interventional Radiology Unit, Hospital Universitari Germans Trias i Pujol, Carretera se Canyet S/N, 08916 Badalona, Spain; 8 National and Kapodistrian University of Athens, Evgenidion Hospital, Athens, Greece; 9 BCLC group Liver Unit, Hospital Clínic-IDIBAPS, CIBEREHD, University of Barcelona, Villarroel 170, 08036 Barcelona, Spain

**Keywords:** Hepatocellular carcinoma, TACE, Chemoembolization, HCC survival

## Abstract

**Purpose:**

To evaluate survival, efficacy and safety of transarterial chemoembolization (TACE) in the treatment of patients with hepatocellular carcinoma (HCC), through a pooled analysis of patients with BCLC 0, A and B HCC stages, treated with polyethylene glycol drug eluting microspheres (PEG-DEM) TACE.

**Materials and Methods:**

Patients from 3 retrospective and 2 prospective registries were included. Overall survival (OS), progression-free survival (PFS), tumour response and safety were evaluated. Multivariate Cox regression analysis was performed to evaluate predictors of OS.

**Results:**

A total of 580 patients (72.1% males, mean age 66.9 ± 10.3 years) were included. 43.5% had BCLC A, and 41.0% BCLC B disease stage, and 85.8% were Child–Pugh class A. Complete and partial response (mRECIST or RECIST1.1) were achieved in 60.14% and 27.11% of patients, with overall response and disease control rates of 87.30% and 94.60%, respectively. Median OS was 50.8 months for the total population, and 61.2 and 38.1 months for BCLC 0 + A and BCLC B patients, respectively. Median PFS for the total population, BCLC 0 + A and BCLC B groups was 15.6, 21.6 and 12.7 months, respectively.

**Conclusions:**

This multicentric pooled analysis confirmed efficacy and safety of PEG-DEM TACE, with a median OS of 50.8 months.

## Introduction

Primary liver cancers, mainly hepatocellular carcinoma (HCC), rank fourth in cancer mortality and sixth in cancer incidence worldwide [1]. Transarterial chemoembolization (TACE) has been established as one of the main treatments for intermediate stage HCC and is currently recommended for treatment of patients with HCC in Barcelona Clinic Liver Cancer (BCLC) stages 0 + A [2] and B [3, 4].

TACE was initially administered in the form of conventional TACE (cTACE), consisting of the intra-arterial injection of an emulsion of lipiodol and a chemotherapeutic agent, followed by embolization of the tumour feeding arteries. More recently, drug eluting microspheres (DEM-TACE) were developed, allowing simultaneous embolization and local release of the pre-loaded drug [5]. Both techniques are widely accepted as first-line treatment of intermediate-stage HCC, nonresectable and non-ablatable HCC in earlier stages, and as a bridging or downstaging treatment before liver transplantation [2–4].

Over the years, better patient selection and application of TACE in a more selective way, aided by imaging, allowed improving its outcomes [6, 7]. Introduction of DEM-TACE allowed for standardized and more consistent delivery of the drug to the tumour [8], contributing to improved DEM-TACE safety [9–14]. Polyethylene glycol drug eluting microspheres (PEG-DEM) have mechanical and drug eluting characteristics similar to other DEM of the same particle size, with hydrophilic properties, can be loaded with a variety of anthracyclines, and remain in suspension for a long time [8]. Early efficacy and safety clinical data for PEG-DEM have been reported [9, 11, 13–17].

The purpose of this multicentric, pooled analysis was to evaluate survival, efficacy and safety of TACE with PEG-DEM loaded with anthracyclines in the treatment of patients with HCC.

## Materials and Methods

### Patient Population and Treatment

The pooled analysis included 5 studies (3 retrospective and 2 prospective) performed between 2015 and 2020, 4 of them published previously [9, 11, 13, 14]. All studies enrolled patients ≥ 18 years old with unresectable HCC that were assigned to TACE by a multidisciplinary tumour board (MDT) according to the institution’s practice.

In one study, doxorubicin dose-escalation protocol was followed [14], while in the other studies, DEM-TACE was performed according to the clinical practice of each participating centre and product instructions for use [18]. All studies used LifePearl™ PEG-DEM (Terumo Europe N.V., Belgium). The choice of microspheres size (100 to 400 µm), treatment schedule, drug dosage (doxorubicin or idarubicin) and follow-up parameters was on the investigator’s discretion in 4 studies or defined per protocol in 1 study.

IRB approval was obtained for three of the datasets in each respective institution, and not required for two of the datasets in each respective institution.

## Outcome Measures

The primary outcome of the analysis was overall survival (OS). Secondary outcomes were safety, efficacy (tumour response evaluated following hospital practice or by independent core laboratory, according to mRECIST (4 studies) or RECIST1.1 (1 study) criteria and analysed as best overall response); progression-free survival (PFS) defined as the first observed disease progression or death; and time to TACE-untreatable progression (TTUP) defined as time to last observed progression after disease control was achieved, as modification of previously described definition [19]. The need and number of repeated TACE procedures for each patient was determined according to clinical decision made by each local MDT.

For all study outcome measures, patients were analysed according to BCLC stage (BCLC 0/A vs BCLC B vs C), the number of lesions and the tumour burden following the “up-to-7” criteria (with 7 being the result of the sum of size in cm and the number of tumours) [20].

## Statistical Methods

Patient demographics, medical history, disease characteristics and procedure parameters are presented as mean ± standard deviation (SD), frequencies and percentages with exact Clopper–Pearson 95% CI for discrete variables.

Results of time-to-event endpoints were estimated using the Kaplan–Meier method with a 95% two-sided confidence interval based on Greenwood formula, with Log Rank *p*-values calculated to test for differences between subgroups. Time to OS was calculated by subtracting the enrolment date from the death date or censor date (the date the subject was last observed and was alive). For PFS, the date of progression or the death date was used as event date. For TTUP, time to event was calculated by subtracting the index date (date of first treatment) from the date of last progression after at least disease control was obtained. Similar calculations were used to calculate time to best response, using the first date the overall best response was reached as the event date. For TTUP, PFS, and best response, times are censored at the last date an imaging was performed. A sensitivity analysis was performed, censoring the times at the date the subject was last observed or the date of resection/transplantation. Patients with BCLC C stage were included in the total population, but, due to the low number of patients in this group, were not included in the sub-analyses.

Cox regression (CoxR), stratified by trial in order to account for between-trial heterogeneity, was performed to predict OS using the following predictor variables: age, gender, Child–Pugh score, ECOG status, liver enzymes, blood and biochemistry parameters, alpha-fetoprotein (AFP), comorbidities, tumour burden, liver segment(s) involved, BCLC stage, hepatobiliary toxicities, number of procedures, dose of anthracyclines and landmark complete and partial response at 3 months. A second Cox regression model was built including only baseline parameters that were not time dependent. To build a multivariable Cox model, univariate CoxR were performed on each predictor variable separately to assess the unadjusted hazard ratios with 95% CI and corresponding p-value. Next, all variables with a univariate *p*-value ≤ 0.20 were added into a multivariable model. Finally, a stepwise Cox or logistic regression was performed on this selection, in which variables with adjusted *p*-value ≤ 0.35 were sequentially entered into the model, whilst being retained if they demonstrated an adjusted *p*-value ≤ 0.20 in the subsequent steps of the stepwise selection. All analyses were carried out using SAS software, version 9·4 (The SAS Institute, Cary NC). All statistical tests were 2-tailed. The analysis between subgroups was exploratory (non-randomized comparisons).

## Results

### Patient Population

A total of 580 patients were analysed, 72.1% of them being male, with mean age of 66.9 ± 10.3 years, Child–Pugh A (85.8%) and BCLC A and B 43.5% and 41.0%, respectively. 61% of the population had multifocal disease (Table 1). Doxorubicin was used in 95.3%, while idarubicin was used in 4.7% of treatments. Mean number of DEM-TACE treatments per patient was 1.89 ± 1.04 (Table 2).

**Table 1:** Patient’s characteristics at baseline

Variable (N of patients in total population with available data)		Total Population	BCLC stage (N)			Up-to-7 Criteria (N)		Number of lesions (N)	
			0 + A (331)	B (238)	C (11)	Within (440)	Beyond (147)	Single (282)	Multiple (227)
Age (mean ± SD, years) (*N* = 580)		66.9 ± 10.3	65.9 ± 10.5*	68.0 ± 9.9*	72.2 ± 9.4*	66.3 ± 10.2*	68.5 ± 10.3*	67.5 ± 10.2	66.8 ± 10.3
Sex, Male (%) (*N* = 580)		72.1%	59.5%*	88.2%	100%*	71.4%	74.2%	72.3%*	88.1%*
Child–Pugh class (*N* = 500)	A	85.8%	87.2%	85.2%	55.6%	85.7%	86.8%	86.1%	85.0%
	B	13.2%	11.5%	14.3%	44.4%	13%	13.2%	12.7%	14.5%
	C	1.0%	1.4%	0.5%	-	1.3%	–	1.2%	0.5%
AFP (mean ± SD), ng/mL (*N* = 443)		687.8 ± 4264.3	512 ± 4867.5*	1002.9 ± 3171.8*	61.5 ± 74.5*	619.7 ± 4701.9*	854.2 ± 2370.6*	265.5 ± 1351.5*	1296.9 ± 6691.7*
AFP > 100 ng/mL (*N* = 454)		24.15%	19.7%	31.3%	25.0%	18.5%*	39.8%*	21.1%	28.8%
Albumin (mean ± SD), g/L (N = 466)		38.1 ± 5.3	38.5 ± 5.3	37.8 ± 5.0	33.5 ± 7.8	38.4 ± 5.2		38.8 ± 5.3*	37.3 ± 5.3*
ALBI Score (mean ± SD)		− 2.5 ± 0.5	− 2.5 ± 0.5	− 2.4 ± 0.5	− 2.0 ± 0.9	− 2.5 ± 0.5	− 2.4 ± 0.6	− 2.5 ± 0.5	− 2.4 ± 0.5
Total bilirubin (mean ± SD), mg/dL (*N *= 549)		1.0 ± 0.7	1.0 ± 0.5	1.0 ± 0.5	2.3 ± 3.6	1.0 ± 0.5	1.1 ± 1.1	1.0 ± 0.9	1.0 ± 0.5
BCLC stage (*N* = 580)	0 – Very early stage	13.6%	23.9%*	–	–	17.8%*	1.4%*	21.1%*	1.3%*
	A – Early stage	43.5%	76.1%*	–	–	50.1%*	23.8%*	45.7%*	33.0%*
	B – Intermediate stage	41.0%	–	100%	–	31%*	70.8%*	31.1%*	63.4%*
	C – Advanced stage	1.9%	–		100%*	1.2%	4.1%	2.1%	2.2%
Tumour characteristics									
Number of lesions (mean ± SD), (*N* = 580)		2.1 ± 1.5	1.9 ± 1.4*	2.4 ± 1.6*	1.8 ± 1.3*	1.7 ± 1.0	3.1 ± 2.1	1.0 ± 0	2.9 ± 1.3
Sum of lesion diameters (mean ± SD), mm (*N* = 509)		52.1 ± 33.3	38.6 ± 23*	69.9 ± 36.5*	79.1 ± 31.8*	37.4 ± 16.8	94.5 ± 33.2	40.1 ± 26.8	64.4 ± 36.2
Multifocal lesions, % (*N* = 580)		61%	42.9%*	62.6%	45.5%*	44.8%	68.7%	0%	100%

*- *p* < 0.05 between the groups (Wilcoxon test for continuous variables, CMH for binary and multinomial variables)

**Table 2:** Characteristics of procedures

Variable	Total		BCLC stage (N)			Up-to-7 criteria (N)		Number of lesions (N)	
			0 + A (331)	B (238)	C (11)	Within (440)	Beyond (147)	Single (282)	Multiple (227)
DEM-TACE number of procedures, *N* = 558									
Mean ± SD Median (Range) Number of TACE treatments		1.9 ± 1.0 2 (1, 7)	1.7 ± 0.9* 1 (1, 5)	2.2 ± 1.2* 2 (1, 7)	1.8 ± 1.3* 1 (1, 5)	1.8 ± 1.0* 2 (1, 6)	2.2 ± 1.2* 2 (1, 7)	1.7 ± 1.0* 1 (1, 7)	2.1 ± 1.1* 2 (1, 7)
	1	44.8%	54.4%	31.2%	54.6%	49.3%	32.6%	53.0%	33.8%
	2	32.1%	29.1%	36.4%	27.3%	30.1%	37.5%	29.1%	35.6%
	3	15.8%	11.7%	21.7%	9.1%	14.4%	19.4%	13.4%	20.6%
	4 or more	7.35%	4.8%	10.8%	9.1%	6.3%	10.4%	4.5%	10.1%
Type of anthracyclines, *N* = 472									
Doxorubicin	95.3%	46.6%	46.6%	2.1%	76.4%	23.6%	52.3%	47.4%	
Idarubicin	4.7%	31.8%	63.6%	4.6%	59.1%	40.9%	30%	70%	
Dose at first TACE (Mean ± SD)									
Doxorubicin, mg		110.8 ± 39.0	103.7 ± 38.7*	117.8 ± 38.0*	112.5 ± 41.3*	107.2 ± 38.8*	122.6 ± 37.1*	108.6 ± 38.6	113.2 ± 39.4
Idarubicin, mg		12.4 ± 4.6	12.9 ± 4.9	12.3 ± 4.8	10.0	11.7 ± 4.4	13.3 ± 5	11.7 ± 4.1	13.4 ± 4.7

*- *p* < 0.05 between the groups (Wilcoxon test for continuous variables, CMH for binary and multinomial variables)

## Survival

**Fig. 1:**
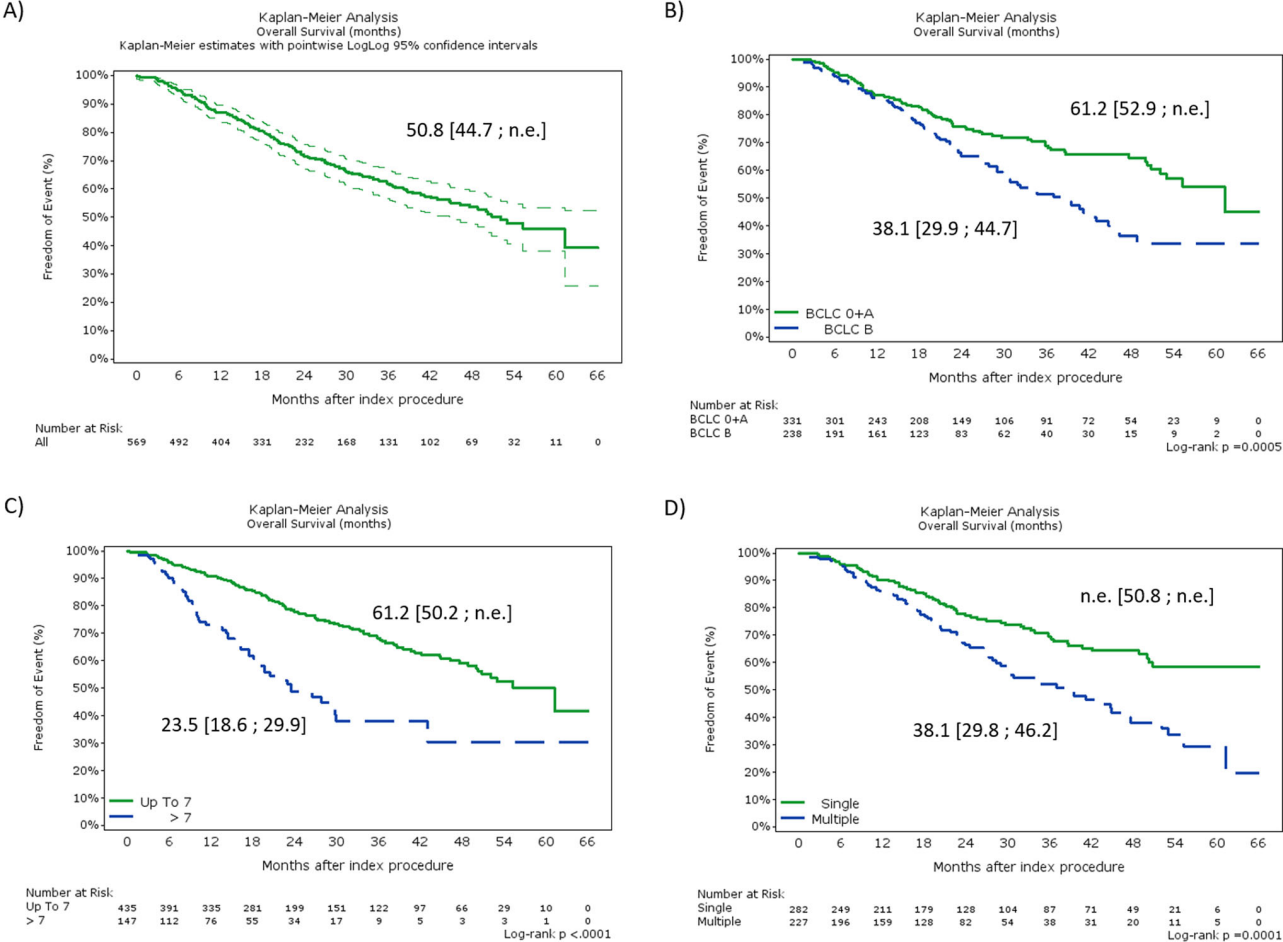
Overall survival (OS) analysis. OS for total population **A**, OS per BCLC stage 0 + A vs B **B**, per Up-to-7 vs Beyond Up-to-7 criteria **C** and per single lesions vs multiple **D**

For the total population, the OS at 12, 18, and 24 months was 86.63%, 80.13% and 71.33%, respectively. Median OS for the total population was 50.8 [44.7; n.e.], for patients with BCLC 0 + A 61.2 [52.9; n.e.] and for patients with BCLC B 38.1 [29.9; 44.7] months (Fig. 1).

A total of 16.9% of included patients were transplanted (*n *= 83) or either transplanted or resected (*n* = 16). When censoring patients at the time of transplantation or resection, median OS for the total population was 42.9 months. When analysed by BCLC stage, it was 52.9 [38.7; n.e.] months for BCLC 0 + A, and 37.0 [29.0; 42.9] months for BCLC B. A total of 35 patients had combined treatment with either thermal ablation or percutaneous ethanol injection to treat persistent tumour.

**Fig. 2:**
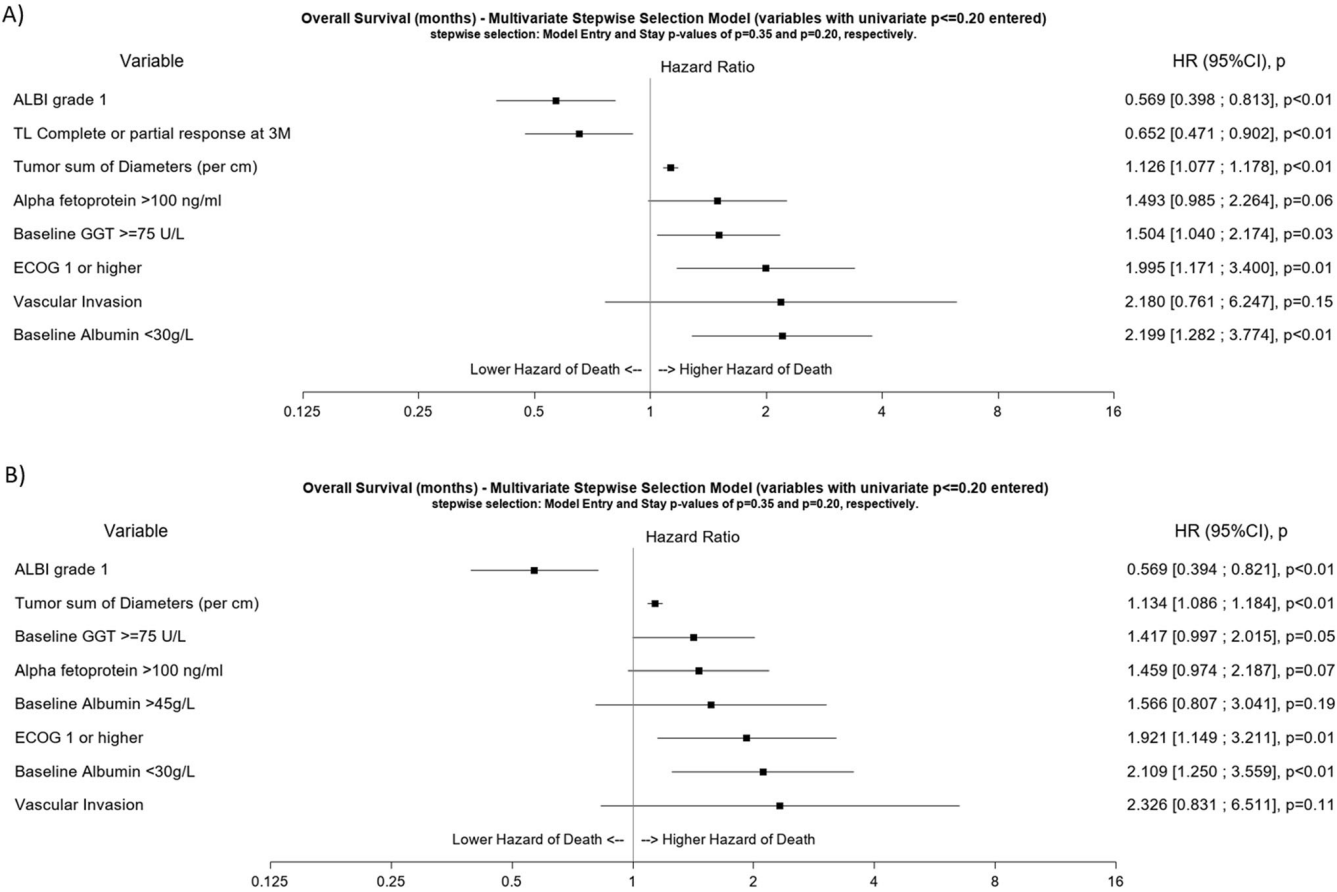
Multivariable Cox regression analysis adding landmark tumour response at 3 months as a predictor of OS **A** and multivariable Cox regression analysis of baseline predictors of OS only **B**

In the Cox regression model including the landmark response at 3 months, identified positive predictive factors for OS were complete or partial response and ALBI score grade 1, whereas baseline albumin of < 30 g/L and a high sum of total tumour diameters were negative predictive factors for OS (Fig. 2A). In the Cox regression model including only baseline parameters, ALBI score grade 1 remained a significant positive predictor of OS, while negative predictors remained the same (Fig. 2B).

**Fig. 3:**
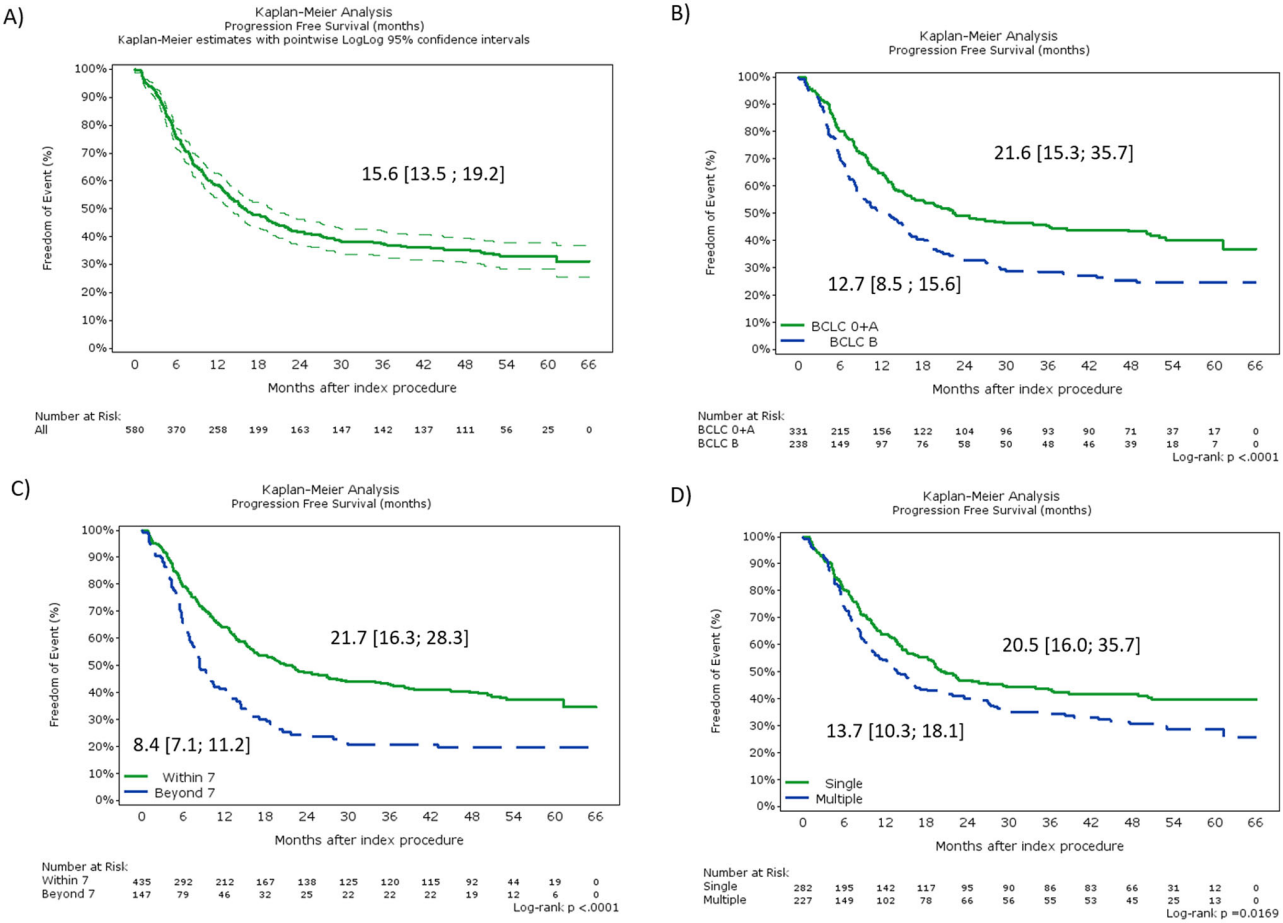
Progression Free Survival. Shown is PFS in total population **A**, PFS per BCLC stage 0 + A vs B **B**, per Up-to-7 vs beyond Up-to-7 criteria) **C** and per single vs multiple lesion (D)

Median PFS for total population, for patients with BCLC 0 + A and B stages were 15.6 [13.5; 19.2], 21.6 [15.3; 35.7], and 12.7 [8.5; 15.6] months, respectively (Fig. 3). The longest PFS was observed in patients which were within the Up-to-7 criteria, with 21.7 [16.3; 28.3] months, while the lowest was in patients outside the Up-to-7 criteria, with 8.4 [7.1; 11.2] months.

Median TTUP was non-estimable (n.e.) for the total population and patients with BCLC 0 + A, while for BCLC B stage it was 17.0 [13.7; 29.9] months. Likewise, median TTUP was non-estimable for patients within “Up-to-7” criteria, while for patients beyond “Up-to-7” criteria, it was 16.0 [10.8; n.e.] months. When stratified by the number of lesions, TTUP was non-estimable for patients with a single lesion, and patients with multiple lesions.

**Fig. 4:**
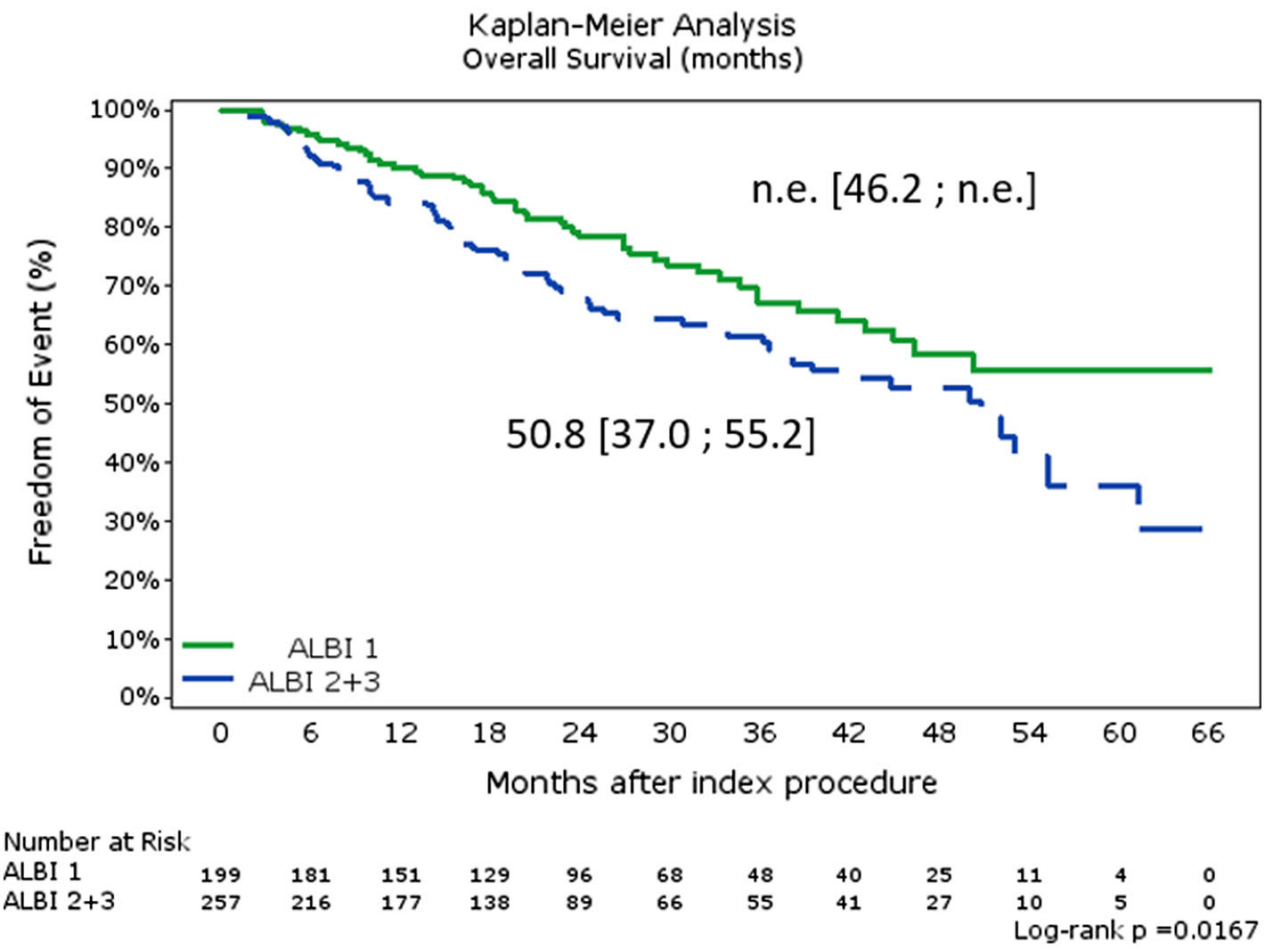
Overall survival (OS) per ALBI score. Shown is OS per ALBI score (1, *N* = 201, and 2 + 3, *N* = 247 for score 2 and *N* = 11 for score 3)

In 456 patients with available ALBI score, TTUP was n.e. [n.e.; n.e.] for patients with ALBI score 1 (*N* = 201) and n.e. [n.e.; n.e.] for score 2 + 3 (*N* = 247 and *N* = 11 for score 2 and 3, respectively). When stratified by ALBI score, median OS was 52.9 [44.9; n.e.] months for all grades, n.e. [46.2; n.e.] months for score 1, and 50.8 [37.0; 55.2] months for score 2/3 (Fig. 4).

## Efficacy

**Fig. 5:**
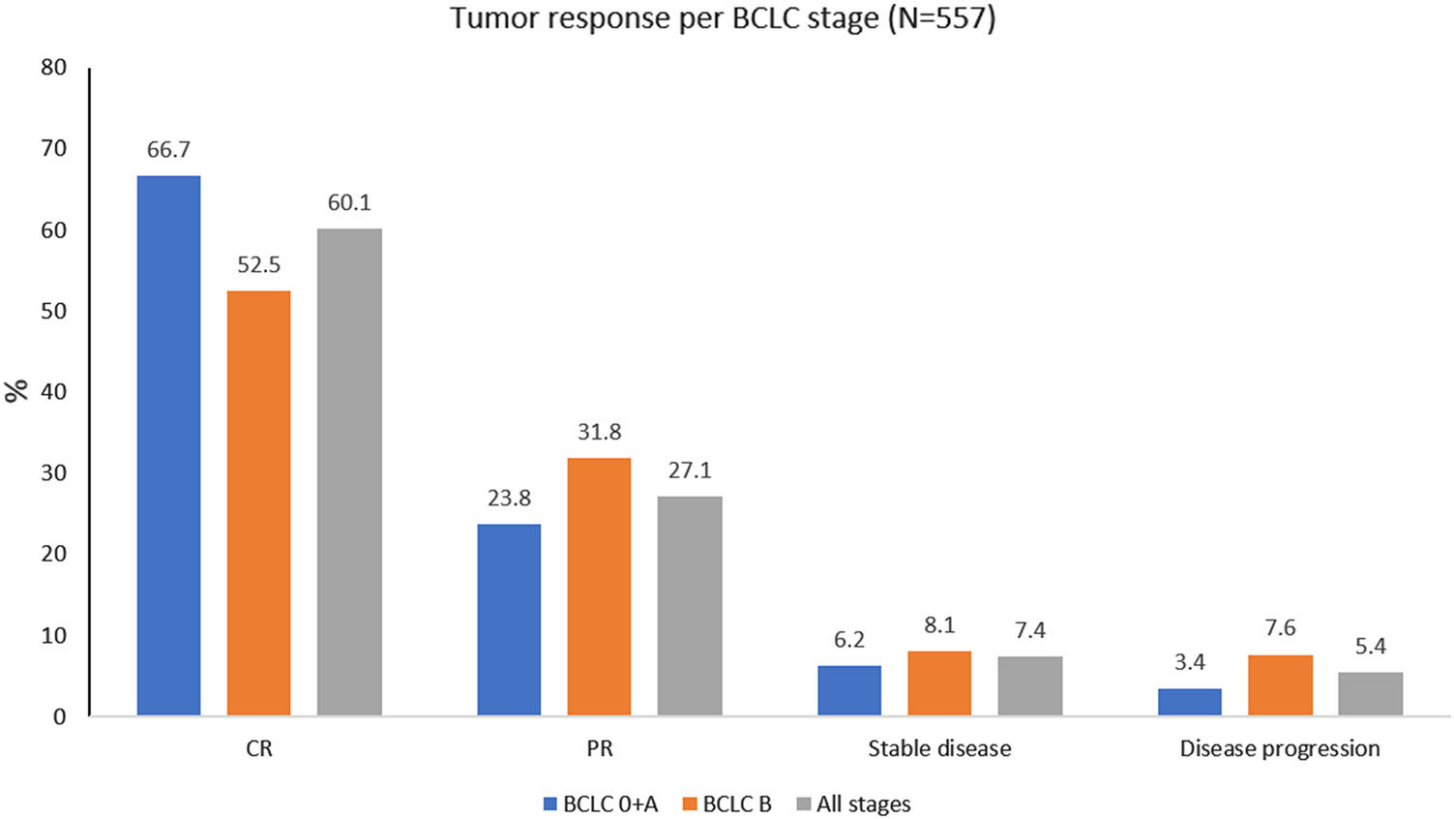
Best overall tumour response per BCLC stage. CR: complete response; PR: partial response

Tumour response data were available for 557 patients. At best tumour response, complete response (CR), partial response (PR) stable disease (SD) and progressive disease (PD) were: 60.14%, 27.11%, 7.36% and 5.39%, respectively, providing an overall response rate and disease control rate of 87.30% and 94.60%, respectively. CR was 60.14%, and when evaluated per BCLC stage, it was 66.67% and 51.07% for patients with BCLC 0 + A, and BCLC B (Fig. 5).

## Safety

Adverse events were reported for 196 patients (33.8%). A total of 2.6% of reported AEs were grade 4, and 1.6% were grade 5, according to the Common Terminology Criteria for Adverse Events (CTCAE). The most frequent AEs were related to post-embolization syndrome (typically characterized by fever, abdominal pain and leukocytosis), which was reported in 4.8% of patients. There were 3 deaths reported within 1 month from the TACE procedure, 2 due to liver failure, who died one month after the procedure, and 1 due to heart failure, who died two days after the procedure. One of the patients who died of liver failure was diagnosed with acute leukaemia at the time of liver decompensation.

## Discussion

This study is one of the largest multicentric cohorts of HCC patients treated with PEG-DEM TACE with doxorubicin or idarubicin to date that suggests a relatively long median OS value. Previous large randomized controlled trials comparing TACE vs TACE combined with targeted agents (sorafenib, brivanib, or orantinib) reported median OS in the TACE-only arm ranging from 19.7 to 33 months [21–24]. As described elsewhere [25] and in accordance with our results, the expected OS in patients in the BCLC B stage was increasing in the last years and may now exceed 30 months [2].

In the Cox regression model with landmark response at 3 months, response to treatment was reported as predictive of survival and remains of potential use as an early surrogate marker if evaluated with appropriate methodology [25, 26]. That may aid decision-making in the multidisciplinary meeting regarding the treatment of HCC patients [27]. Performance status ≥ 1, low baseline albumin and sum of tumour diameters were identified as negative survival predictors, confirming previously reported results [28].

More than 60% of patients in the current analysis achieved an imaging CR after one or more TACE treatments. This level of CR is clearly exceeding what is reported in TACE 2 (23%) [23], SPACE (14.1%) [29], and TACTICS (27.6%) [24] trials, and can potentially explain the observed longer OS [30]. The large proportion (more than 50%) of patients with early-stage HCC (BCLC 0 + A) certainly contributed to the OS, however, even patients in BCLC B survived longer (median OS 38.1 months) than in other studies that included between 27 and 44% of the patients in an early stage.

Despite the difference in definition, in the present study, median TTUP was non-estimable, and even in BCLC B patients it was close to what has been reported in the TACTICS trial, where TTUP was 20.6 months, for a population which included 44% of BCLB A patients, in the TACE-only group [24]. In that analysis, the treating physician made decision to discontinue TACE treatment, and some patients were switched to other therapies without reaching *unTACEable* (untreatable by TACE) progression [24]. Moreover, 17% of patients included in that analysis have been transplanted or resected, therefore reducing TTUP due to censoring. In our analysis, TTUP in patients with baseline ALBI score 1 and 2 + 3 was not different. Since 44% of patients received two or more TACE (range 2–7), that might suggest that reaching unTACEeable progression was more related to tumour progression rather than liver function deterioration. This is supported by the TACTICS trial, where only 4 out of 99 patients who reached TTUP, did so due to liver function deterioration, with 95 showing TACE refractoriness or tumour progression as the reason for reaching TTUP [31]. These findings contrast with what had been previously reported in the SPACE trial, with TTUP being reached due to liver function deterioration in 42.7% treated with DEM-TACE and placebo, versus 61.8% treated with DEM-TACE combined with Sorafenib, reflecting the difference in definitions of TTUP in the two trials [30].

Our findings, obtained before immunotherapy became part of the standard of care for HCC, can serve as a landmark for expected OS in the era of combining TACE with systemic therapies, including immune therapies [31].

Regarding safety of TACE, our 33.8% global rate of complications was mostly related with mild post-embolization syndrome with only 2.6% grade 4 AEs and 1.6% of grade 5. Image findings of hepatic damage occurred in 12.6% of patients [9], which is lower than the 30.4% and 36.8% previously reported for DEM-TACE, and in accordance to that reported with cTACE [32, 33].

In Europe, TACE is actively used for bridging patients to liver transplantation, also contributing to prolonged survival after transplantation [34]. Even if the natural history of HCC is inextricably linked to the underlying liver disease, the improvement in OS observed in BCLC intermediate stage patients from 30% 3-year OS in 2002 to 60% in 2012 [35] could largely be attributed to improvements in TACE technique and patient selection, with implementation of imaging methods and identification of a patient cohort that can benefit most from TACE. Since then, TACE filled the place of a therapy of choice for intermediate HCC with expected survival of > 2.5 years [2].

Our study has limitations, mainly due to the pooling of diverse studies. Data collection, imaging time intervals and follow-up were not standardized across the studies, and patient populations varied between the study sites, which may all compromise the predictive power in the CoxR models. Tumour response was measured according to mRECIST in 4 studies and RECIST1.1 in one study (which provided 50 patients), which may have influenced the response analysis. The survival analysis may have been biased by the inclusion of 35 patients who were treated with thermal ablation or percutaneous ethanol injection for persistent tumour. Another limitation of the study was incompleteness of data that did not allow for time dependent analysis of survival predictors. Despite these limitations, we found it important to evaluate tumour response as predictor in the landmark model and the obtained results suggest that it can be used as surrogate predictor of survival in similar studies [25, 26]. Future studies on TACE, either alone versus systemic therapy or in combination with systemic therapy will redefine the treatment of HCC, and our data may provide a background to select the target population and the baseline assumptions of such studies. Further prospective studies will focus on combinations of TACE with systemic therapies and are currently being explored.

## Conclusions

This multicentric pooled analysis of 580 patients with HCC, treated with PEG-DEM TACE, demonstrated a median OS of 50.8 months, contributing to the knowledge of the current expected survival after TACE, for the treatment of HCC.
